# Cyclosporine Regimens in Plaque Psoriasis: An Overview with Special Emphasis on Dose, Duration, and Old and New Treatment Approaches

**DOI:** 10.1155/2013/805705

**Published:** 2013-07-25

**Authors:** M. D. Colombo, N. Cassano, G. Bellia, G. A. Vena

**Affiliations:** ^1^Novartis Farma S.p.A., Origgio, Varese, Italy; ^2^Department of Dermatology, Marchesi Hospital, Inzago, Milan, Italy; ^3^Dermatology and Venereology Private Practice, Bari and Barletta, Italy

## Abstract

Cyclosporine A (CsA) is one of the most effective systemic drugs available for the treatment of psoriasis, as evidenced by the results of several randomized studies and by a prolonged experience in dermatological setting. In clinical practice, CsA is usually used for the induction of psoriasis remission at a daily dose included in the range of 2.5–5 mg/kg and with intermittent short-term regimens, lasting on average 3–6 months. The magnitude and rapidity of response are dose dependent, as well as the risk of development of adverse events. Therefore, the dose should be tailored to patient's needs and general characteristics and adjusted during the treatment course according to both the efficacy and tolerability. Some studies support the feasibility of pulse administration of CsA for a few days per week for both the induction and the maintenance of response in psoriasis patients. This paper will review the data on CsA regimens for plaque-type psoriasis and will focus the attention on dose, treatment duration, novel schedules, and role in combination therapies, including the association with biologicals.

## 1. Introduction

Psoriasis is a chronic inflammatory immunomediated disease of unknown aetiology, which is significantly associated with psychological distress and impaired quality of life [1–3]. Treatment of this condition is not curative but is aimed at inducing a temporary control of clinical manifestations and improving the impact of the disease on quality of life and the level of acceptance of the disease.

The management of a chronic disease like psoriasis is complex and is conditioned by multiple factors, including, but not limited to, the objective severity and distribution of skin lesions, the influence on psychosocial aspects, the response to previous therapies, and the presence of concomitant psoriatic arthritis (PsA) and comorbidities. Therapeutic management of psoriasis usually requires a patient-tailored approach in which combination and sequential therapies are often considered over time in order to augment response, to optimize the safety profile, and/or to meet specific clinical needs. There is a wide armamentarium of therapeutic tools available for the treatment of psoriasis which includes topical medications, phototherapy, and systemic nonbiological and biological drugs. 

It is estimated that moderate-to-severe psoriasis accounts for about 25% of psoriasis patients [1], most of whom are likely to require systemic drugs or phototherapy. When psoriasis requires systemic therapy, cyclosporine (CsA) is one of the most effective and rapidly acting drugs. 

Since the time of the first observations documenting the clinical activity of CsA in psoriasis, more than 30 years ago [4], a considerable amount of clinical data has been accumulated in favour of the efficacy and safety of the drug in many immunomediated skin disorders and especially psoriasis and atopic dermatitis. In light of the current knowledge and after several years of experience gathered in clinical practice, CsA is often used as first-line therapy in moderate-to-severe forms of psoriasis by several dermatologists [5]. Psoriasis treatment regimens with CsA have to be adapted to the patient's needs and specific characteristics, after an accurate selection and a careful assessment of the risk/benefit ratio.

This paper was intended to review the information currently available on CsA regimens for plaque-type psoriasis, focusing the attention on dose, treatment duration, novel schedules, and role in combination or rotational therapies.

## 2. Dose of CsA

### 2.1. General Data

In dermatological practice, the daily dose of CsA is usually in a therapeutic range of 2.5–5 mg/kg. The use of such doses for a short-term course (12–16 weeks) has been shown to cause a rapid and significant improvement or complete remission in 80–90% of psoriasis patients [6]. Exceeding the dose of 5 mg/kg/day does not yield any additional benefit in terms of efficacy in psoriasis, whereas it notably increases the risk of side effects [7]. The higher is the dosage, the better and quicker are the results of treatment. At doses of 4-5 mg/kg/day, CsA is a very active drug, characterized by a rapid onset of response, as demonstrated by the extent and speed of reduction in the Psoriasis Area and Severity Index (PASI) score. A dose of 2.5–3 mg/kg/day has a better risk/benefit ratio, with attainment of the highest efficacy in approximately 2-3 months [8]. The efficacy of CsA in plaque psoriasis has been evidenced by several randomized studies, which also showed the dosage-dependent therapeutic effects, using the drug at dosages ranging from 1.25 to 5 mg/kg/day for 10–16 weeks on average for the induction of psoriasis remission. The most important randomized trials are summarized in Table 1 [7, 9–17]. 

A meta-analysis of 3 major randomized studies [9, 11, 13] involving 579 patients with severe psoriasis revealed that, after 10–12 weeks of CsA treatment at doses of 1.25, 2.5, and 5 mg/kg/day, there were PASI reductions of 44.4%, 69.8%, and 71.5%, respectively. The average time needed for the achievement of at least 50% of PASI reduction from baseline (PASI 50) was 4.3 weeks for 5 mg/kg/day, 6.1 weeks for 2.5 mg/kg/day, and 14.1 weeks for the lowest dose [18]. 

Undoubtedly, the choice of the initial dose is not only dependent on the personal experience of the dermatologist, but also on the cutaneous and general conditions of the patient, taking into account the strong influence of the dose on both the clinical response, in terms of either speed or magnitude, and the risk of adverse effects [7, 12, 15]. Whereas good general conditions exist, it is advisable to start with a low-dose regimen in patients with stable and less severe psoriasis and to start with a full-dose regimen in case of severe recalcitrant and unstable forms, or whenever rapid control of the disease is required. Some authors prefer to start at full dosages until the achievement of remission and then gradually taper the dosage, adjusting it only in case of adverse reactions (step-down regimen). Others, conversely, advise to start with daily doses of 2.5–3 mg/kg and gradually build up the dose by 0.5–1 mg/kg/day every 2–4 weeks in the event of nonresponse, carefully monitoring tolerability (step-up regimen) [8, 15, 19, 20]. 

The outcomes of such different regimens were analysed by a 12-week, prospective, open-label study in 61 severe psoriasis patients [21]. Patients were assigned to a 2.5 mg/kg/day starting dose and an increasing “step-up” regimen or a 5.0 mg/kg/day starting dose and a decreasing “step-down” regimen group. The PASI 50 response rates at 12 weeks were 72.7% and 85.7% for the step-up and step-down regimens, respectively, but this difference was not statistically significant. Instead, a PASI improvement of 75% or more (PASI 75) at 12 weeks was significantly higher with the step-down regimen as compared to the other regimen (75.0% versus 51.5%). The mean time to reach PASI 75 in the step-down regimen was significantly shorter than that in the increasing dose regimen (5.8 weeks versus 7.8 weeks). 

As with other pruritic skin diseases, the relief of pruritus with CsA is generally marked and rapid, especially when higher doses are used [5, 22]. Pruritus is often complained by psoriasis patients and sometimes reported as unbearable, although it has been underestimated for a long time. This symptom should not be neglected because it can be a source of psychological distress and in turn may worsen the skin lesions through the “Koebnerization” induced by scratching. Clinical response is coupled to a significant improvement in quality of life parameters [23]. 

### 2.2. Novel Findings: Low Dosages, Fixed Dose, and Preprandial Intake

Although the therapeutic range of CsA is described to correspond to 2.5–5 mg/kg/day, with 2.5 mg/kg/day reported as the optimal starting dose in most cases, it is established that lower doses may be also effective, with satisfactory effects attainable at least in a subset of psoriatic subjects [11]. A dosage of 1.25 mg/kg/day CsA has been found to be superior to placebo [18]. Therefore, it is not surprising that, in clinical practice, CsA is often used at low dosages, of 3 mg/kg/day or less, and sometimes lower than those comprised in the conventional therapeutic range and that, even at low dosages, the effectiveness of CsA is often remarkable in the routine management of psoriasis.

In a retrospective analysis of 193 patients, CsA was administered for a mean period of 14 months, for 1–4 courses (mean: 1.6), and the mean dosage ranged from 1.5 to 3.1 mg/kg/day [24]. The average PASI reduction at the end of treatment from baseline was 76%. The PASI 50 was achieved in 91.3% of patients and the PASI 75 in 73.9%. 

The efficacy of 3 mg/kg/day administration of CsA was investigated in 35 patients who were treated until the PASI 75 was reached. Remission was achieved in 26 patients (74%) after a mean period of 101.5 days [25]. In this study, CsA was taken twice daily before breakfast and dinner. 

The information about drug intake before or after meals is rarely mentioned in publications regarding CsA on psoriasis. Therefore, it is not possible to draw considerations regarding the correlation between efficacy and preprandial or postprandial intake of CsA. CsA is usually administered after meals. The advice to take the drug after meals traditionally applies to the old formulation, which had its bioavailability increased thanks to the intake after a fat-rich meal. The new CsA microemulsion has improved the absorption profile substantially, increasing bioavailability and reducing pharmacokinetic variability, and this leads to a more consistent clinical response at a given dose [14, 26]. 

Preprandial administration of CsA has been recently shown to enhance drug absorption in patients with nephrotic syndrome [27]. This finding has been replicated in psoriasis patients, in whom the preprandial administration of CsA was found to enhance drug absorption [28], thus allowing the dose to be reduced and resulting in more reliable pharmacokinetics. Clinical evidence also confirmed a greater efficacy of preprandial intake of CsA. In fact, in an open trial, 37 patients were randomly assigned to receive CsA microemulsion 1.5–3.0 mg/kg/day before or after meals, once daily for up to 6 weeks [29]. The percent reduction in PASI from baseline was 75.4% in the former group and 29.8% in the latter. 

A recent interesting study in 61 obese patients with moderate-to-severe psoriasis evaluated the effects of low-dose CsA (2.5 mg/kg/day) alone or in association with a calorie-controlled diet leading to significant weight loss (mean reduction in body weight of 7% at week 24) [30]. The PASI 75 response was achieved by 66.7% of subjects treated with CsA plus a low-calorie diet and by 29% patients treated with CsA alone. 

Body-weight-dependent (BWD) dosing of CsA is generally recommended. The motivation for BWD dosing is the potential for renal impairment which shows a clear dose relationship, although there is a weak correlation between weight and increased nephrotoxicity with conventional BWD dosing of CsA in psoriasis patients, especially in long-term treatment [10, 31]. Some studies tried to examine the effects of a fixed dose which can be more practical in clinical setting. A randomized parallel-group study in adults with severe psoriasis (Table 1) compared daily CsA doses of 100–300 mg, given in a body-weight-independent (BWI) manner, or 1.25–5.0 mg/kg (BWD) for 12 weeks. The initial dose of CsA was 200 mg daily in the BWI regimen and 2.5 mg/kg/day in the BWD regimen, with the possibility of stepwise adjustments (ranges of 100–300 mg and 1.25–5 mg/kg/day for the BWI and BWD dosing, resp.). The efficacy of regimens was similar, with a mean decrease in PASI of 86% in the BWI group and 87% in the BWD population, and this was achieved with a mean final dose of approximately 230 mg in both groups [10]. 

Another randomized study evaluated whether a fixed-dose CsA microemulsion of 100 mg/day is effective for treating psoriasis [32]. Forty patients were given either 100 mg CsA once daily (group A) or 50 mg twice daily (group B), regardless of patient weight and condition. Also on this occasion, the drug was taken before meals. Mean body weights were 66.7 and 68.6 kg, respectively. At 12 weeks, PASI improvement rate was 69.4 in group A and 73.4 in group B, whereas PASI 50 was achieved by 82% in group A and 84% in group B. At 6 weeks, the number of patients with PASI 50 was significantly higher in group A than in group B.

## 3. Duration of Treatment

Short-term treatment (4–8 weeks) with CsA may be useful to obtain rapid control of particularly severe forms, such as generalized pustular psoriasis, thanks to the rapid onset of action of the drug [33]. In general, after resolution of acute flares following short-term treatment, most cases can be gradually managed by conventional treatments afterwards [8, 34]. In clinical practice, for the management of uncomplicated cases of moderate-to-severe plaque psoriasis, CsA is generally used for induction of remission with intermittent short courses generally lasting up to 24 weeks [5], discontinuing the drug after complete remission is achieved. Various attempts have been made to maintain remission in psoriasis patients treated with continuous CsA, such as reduction in daily dose, intermittent CsA dosing, or switching to topical therapy. In case of relapse, patients may undertake a new cycle using the last most effective and best tolerated dose of CsA [19].

Once clinical remission is obtained, it should be decided whether treatment has to be suddenly stopped or gradually tapered up to a maintenance minimum effective dose. At any rate, the goal of maintenance therapy is not necessarily the complete disappearance of lesions, but rather to make the disease tolerable to patients, while avoiding a superfluous and potentially harmful overtreatment. Gradual tapering avoids early relapses, although abrupt suspension of CsA is not associated with rebound phenomenon [35–37].

Relapses seem to develop later and less frequently in those patients who experience a complete clinical remission compared to those who show only a partial improvement at the end of treatment [19]. The median time to relapse was found to become progressively shorter after multiple treatment courses [37]. Duration of remission appears to be also conditioned by baseline severity. 

In fact, in a recent study [25], after drug withdrawal in PASI 75 responders, the average length of time before restarting systemic therapy was found to be 182 days, ranging from 120.1 days for patients with PASI scores of 13 or more to 287.5 days for patients with PASI scores of <13. 

The PISCES study also compared the effects of abrupt against gradual discontinuation of CsA [6]. A total of 45% of subjects had not relapsed 4 months after stopping treatment, and 31% had not relapsed after 6 months. Median time to relapse was 109 days in the patients who abruptly stopped CsA and 113 days for patients who were tapered off. However, Hakkaart-van Roijen et al. [38] showed that gradually tapering the CsA dose before discontinuing treatment results in both lower costs and improved efficacy in comparison with abrupt discontinuation, improving the overall mean incremental cost-effectiveness ratio. These results corroborated previous findings showing better preservation of remission by drug tapering rather than abrupt discontinuation [13]. 

Longer-term continuous therapy may be required for maintenance in a minority of patients with recalcitrant disease, often with doses less than 3.5 mg/kg/day [34]. However, renal dysfunction related to long-term CsA maintenance therapy is a major concern. While intermittent short courses are associated with dose-dependent, transient, and reversible renal function abnormalities, renal structural alterations have been demonstrated in a small percentage of patients after 2 years of continuous treatment at 5 mg/kg/day [37, 39, 40]. Within this period, once the drug is withdrawn, the nephropathy is not progressive. For this reason, the US and European guidelines recommend to avoid continuous treatment for more than 1 year and 2 years, respectively [34, 40–43].

Table 2 summarizes some of the most important studies exploring long-term management with CsA after induction of psoriasis remission (e.g., intermittent or continuous treatment strategies or maintenance treatment) [6, 12, 36, 37, 44–47].

## 4. Pulse Treatment 

Some studies explored the feasibility of CsA treatment as pulse administration for a few days per week for the induction or maintenance of psoriasis remission.

### 4.1. For Induction of Remission

Starting from the premise of the 36-hour cell cycle of psoriatic keratinocytes, more than 20 years ago, Goodman et al. [48] studied the effects a new CsA regimen in 15 psoriasis patients, consisting in a 36-hour weekly schedule. This “cell-cycle-derived dosing schedule” was probably based on the traditional regimen with methotrexate for psoriasis. In particular, CsA was administered at 12-hour intervals for three consecutive doses per week for 10 weeks. The initial dose was 2.5 mg/kg/dose and was then increased every 2 weeks by 2.5 mg/kg/dose reaching a maximum of 10 mg/kg/dose. PASI 50 and PASI 75 responses were observed in 60% and 40% of cases, respectively, with a total PASI reduction of 61% detected in the total patient series. However, not surprisingly, the use of such extremely high CsA dosages was associated with relevant side effects, leading to permanent treatment discontinuation in 20% of patients and continuation of treatment at reduced doses in further 20% of cases. 

An alternative pulse regimen was evaluated in a pilot study in 203 patients with moderate-to-severe plaque psoriasis (PASI score of at least 12). Patients were allocated to receive CsA 4 mg/kg/day, taken every day for 6 months (*n* = 101) or CsA at the same daily dosage administered for 4 consecutive days for 6 months (*n* = 102) [49]. At baseline, more patients in the pulse treatment group had borderline or stage I hypertension as compared to patients enrolled in the continuous daily treatment arm (47 versus 25 subjects, resp.). However, the development of relevant abnormalities in blood pressure was 3-fold less frequent in patients treated with CsA for 4 days per week. Daily treatment caused the achievement of the PASI 50 and PASI 75 at 2 months in 60% and 14% of cases as compared to 43% and 8% in the other group, respectively. The PASI 75 response rates at 4 months and 6 months were 60% and 84% in patients who took CsA every day and 47% and 78% in those under the intermittent treatment, respectively. Therefore, the “4 on/3 off” regimen was associated with a slower onset of action, but at 6 months differences in efficacy between treatment groups appeared unremarkable. 

### 4.2. For Maintenance of Response

A randomized double-blind placebo-controlled extension phase enrolled patients who had reached the PASI 75 after the first phase of the study [10]. These patients were rerandomized to receive placebo (*n* = 51) or CsA (*n* = 42) at their last effective 3 times weekly for 12 weeks. Relapse (defined as an increase in PASI to more than 50% of baseline value) occurred in 40.5% of cases with intermittent CsA and 56.9% with placebo (*P* = 0.15). The relatively short follow-up duration might have influenced such results. The time to relapse was calculated as 98 days under intermittent CsA and 69 days under placebo. High rates of treatment failure were registered in the placebo group (41.2%) compared with the CsA group (23.3%), leading to significant differences in the rate of study discontinuation between groups.

In an open-label trial, 46 psoriasis patients received a maintenance therapy with 5 mg/kg/day every 4 days after an induction phase with CsA 5 mg/kg/day for 4 weeks [50]. Over a treatment period of 3 to 6 months, complete response was achieved by 12 patients (26%), marked improvement still persisted in 26 patients (56.5%), whereas psoriasis relapsed in 8 subjects (17.5%).

Another report evaluated the feasibility of a maintenance regimen with 5 mg/kg CsA twice weekly in 11 psoriasis patients [51]. These patients were initially treated with 5 mg/kg/day for 1-2 months until clearance of lesions. Maintenance treatment with CsA 1.5–3 mg/kg/d for 2–4 months proved to be effective in controlling psoriasis but was associated with side effects which were poorly tolerated even if they were usually mild. An alternative maintenance regimen with 5 mg/kg/day twice weekly led to a good control of psoriasis without relapse and with a modest irrelevant PASI increase (mean: 5%), displaying a good tolerability profile.

This pilot experience inspired a larger randomized controlled study named Psoriasis Relapse Evaluation with Week-End Neoral Treatment (PREWENT) study, aimed at evaluating the efficacy and tolerability of week-end CsA microemulsion for reducing relapse rate in patients with chronic plaque psoriasis who had achieved clinical remission following continuous CsA therapy [52]. The primary endpoint was clinical success rate at week 24 (no relapse or a PASI <75% of pretreatment PASI). Patients were randomized to CsA (*n* = 162) or placebo (*n* = 81) for two consecutive days per week for 24 weeks. Clinical success rates at 24 weeks were 66.9% and 53.2% with CsA and placebo, respectively (*P* = 0.072). Time to first relapse was significantly prolonged with CsA versus placebo (*P* = 0.023), and PASI was significantly lower from weeks 4 to 16 in CsA recipients. In patients with moderate-severe psoriasis, clinical success rate was significantly improved with CsA (69.9% versus 46.3%; *P* = 0.011), and significantly lower increases in PASI were observed from week 4 to week 24 (*P* < 0.05 versus placebo). In agreement with previous results [6, 35, 36], patients who interrupted treatment did not exhibit psoriasis rebound. 

A post hoc subgroup analysis of the primary endpoint was also performed to estimate the difference between treatment groups according to disease severity at entry before continuous induction therapy with CsA [50]. Stratifying patients by pretreatment PASI score tertiles, it became clear that, in patients belonging to tertiles I and II (thus excluding the most severe patients), week-end CsA therapy was significantly more effective than placebo during the whole 6-month maintenance phase, whereas patients with very severe disease (tertile III) showed a progressive increase in PASI scores with CsA week-end treatment similar to that observed with placebo. In parallel, relapse rate following 6-month CsA week-end treatment was 30.1% in patients included in the tertiles I and II as compared to 53.7% in placebo recipients (*P* = 0.01). CsA was well tolerated, with no differences in mean blood creatinine or blood pressure observed between CsA and placebo. Therefore, week-end CsA administration was shown to prolong safely and effectively the time to first relapse in psoriasis patients [52].

## 5. Combination and Rotational Therapy

CsA effects usually persist over a period of 2-3 months after drug withdrawal, allowing the use of intermittent therapy. During treatment-free intervals, topical therapy may be prescribed as needed to control localized lesions. Alternatively, sunlight exposure may help keeping remission, as it often happens at our latitude [5]. 

Long-term management of psoriasis requires an individualized approach. Rotational and combination treatments are practical strategies commonly used in clinical setting to reduce the cumulative toxicity of antipsoriasis treatments and to optimize their risk/benefit ratio. Because of its high and rapid efficacy, CsA rarely needs to be associated with other systemic therapies, with the exception of selected cases. Anyway, combinations which are contraindicated are CsA and phototherapy with both UVB and PUVA, while combined use of methotrexate-CsA and CsA-acitretin requires careful monitoring and might be helpful in patients with severe and recalcitrant psoriasis [53]. 

The concurrent administration of CsA and UVB has not been studied extensively and, even if contraindicated, has successfully been used in sporadic cases for a short period of time [54]. While a recent systematic review with over 25 years of dermatologic experience worldwide does not clearly substantiate that skin cancer risk is necessarily increased in patients using CsA for cutaneous diseases, unlike organ transplant recipients [55], it is well established that there is an increased risk of nonmelanoma skin cancers with association of PUVA therapy and CsA [56]. In a comparative, open-label study, narrow-band- (NB-) UVB phototherapy alone was compared with sequential CsA-NB-UVB in two groups of 30 patients with plaque psoriasis (PASI > 15). In the latter group, 3 mg/kg/day CsA was administered for 4 weeks and then was rapidly tapered while phototherapy was started. Treatments were given until psoriasis cleared or until partial improvement was observed without further amelioration after another week of therapy. The two treatments attained similar efficacy, but, in the sequential protocol, the short-term use of CsA allowed lowering of the total NB-UVB doses and the cumulative number of exposures [57]. 

Due to its prompt effectiveness and rapid onset of action, CsA is considered an “accelerator” of clinical response, unlike other slow-acting molecules, that is, acitretin, which are instead considered “maintainers.” CsA can be therefore used first as a clearing agent with subsequent acitretin as maintenance therapy [58]. Based on these premises, a well-known sequential regimen suggests the initial use of CsA monotherapy, and, once psoriasis control is obtained, acitretin is introduced, while CsA is gradually tapered, and then discontinued. Acitretin can be then used as monotherapy for long-term maintenance [59]. In such a rotational scheme, as with combination, an advantage is retinoids' possible limitation of development of tumoral and pretumoral skin lesions. The compatibility of concurrent treatment with CsA and oral retinoids was first documented in transplant patients using acitretin to control skin complications. No unpredicted adverse effects were noted. Nevertheless, short-term use of this approach is advisable [60]. Lipid profiles must be closely monitored because both retinoids and CsA may increase serum cholesterol and triglyceride levels. No metabolic interaction has been demonstrated between CsA and etretinate in vitro [61]. Some reports in psoriatic patients however showed controversial results of combination of CsA and oral retinoids [62–64].

The concomitant administration of methotrexate and CsA has been successfully used for the treatment of rheumatoid arthritis and PsA [65–67]. In general, this combination is commonly considered more in rheumatological practice, using relatively low dosages of both drugs, than in dermatological setting. The combination of CsA and methotrexate reduces the dosages and also the side effects of each agent, allowing better disease control with less toxicity [68]. A 12-month, randomised, double-blind, placebo-controlled trial in 72 patients with active PsA with a partial response to methotrexate showed that combining CsA and methotrexate treatment significantly improves the signs of inflammation [69]. Methotrexate combined with CsA is also effective in psoriasis, including recalcitrant generalized pustular forms. Combination of 7.5–15 mg/week of methotrexate with 3 mg/kg/day of CsA was found to produce better clearance of psoriasis and fewer side effects than monotherapy with either agent [70]. Although no controlled studies have been performed, other reports support those conclusions [60]. In a more recent prospective study [71], 20 patients with severe psoriasis had clinically significant improvement after treatment with the combination of methotrexate, given intramuscularly as a single weekly dose of 10 mg, and CsA at a dose of 3.5 mg/kg/day, for a median period of 9.5 weeks (range 4–50). Short-term side effects were minor, transient, and manageable. A retrospective study examined the effects of CsA associated with methotrexate in 18 patients with moderate-to-severe psoriasis, 14 treated with short-term and 4 with long-term combination therapy [72]. Twelve patients in the first group and all patients of the second group achieved the PASI 50. Nine patients in the first group and all patients in the second group suffered from significant but reversible adverse effects. Adequate monitoring of tolerability was therefore recommended by the authors. Extreme caution was suggested by other authors [73] due to the risk of the reduced clearance of each drug induced by the other. This interference might be responsible for increased blood concentrations of both drugs and subsequent toxic effects, that is, increased serum creatinine and transaminases, observed in 4 psoriasis patients. 

Interestingly, a synergistic and successful activity of methotrexate-CsA combination was described in a patient who had his psoriatic skin lesions not controlled by methotrexate alone and his arthritis symptoms not controlled by CsA alone [74].

As concerns the combination of CsA and topical drugs, which can be safely used to augment and accelerate the responsiveness to CsA, especially in regimens with low-dose CsA or while CsA dosage is tapering off, evidence exists for anthralin and calcipotriol alone or calcipotriol associated with betamethasone dipropionate as two-compound product (CBD). The association of topical medications may contribute to spare the cumulative exposure to the systemic drugs on the long term.

CsA and dithranol used concurrently produce benefits in a subset of patients described by Gottlieb et al. as “slow responders” [75]. In a right-left comparison study, the subset had a significantly lower severity index and less histopathological changes at lesional sites on the anthralin-treated side at 18 weeks of treatment. In a double-blind placebo-controlled study, CsA at a daily dose of 2 mg/kg for 6 weeks in association with a placebo ointment caused a reduction of 57.7% in the mean PASI, while a decrease of 80.5% of the PASI was observed when calcipotriol ointment was added to the same dose of CsA for 6 weeks [76]. In the CsA-calcipotriene ointment combination group, psoriasis clearing was obtained at 6 weeks in 50% of patients versus 11.8% of patients treated with CsA alone. Similar results were seen using low-dose CsA combined with CBD ointment [77]. In a randomized open-label study, 60 patients with moderate-to-severe plaque psoriasis were allocated to receive CsA, 2 mg/kg/day, combined with CBD ointment or CsA, at the same daily dose, in combination with an emollient, for 8 weeks. Combination therapy with CsA and CBD ointment was more effective than CsA and emollient treatment, with statistically significant results, particularly less itching after 4 and 8 weeks and PASI reduction at all postbaseline visits. Significantly more patients treated with CsA plus CBD achieved the PASI 75 at 8 weeks (87% versus 37% in the CsA-emollient group). 

Combination of CsA with systemic biological or nonbiological therapies should be reserved to particularly severe recalcitrant cases of psoriasis and only for limited periods of time. Combined therapy with systemic biological and traditional agents is not generally indicated for psoriasis treatment, although it is increasingly used for the treatment of “high-need” patients with psoriasis. There are only limited data on the combination of CsA with biological drugs for the treatment of psoriatic disease [78]. CsA has been administered as a rescue option, with or without interruption of biological therapy, in patients experiencing transitory return or severe exacerbations of psoriasis lesions during biological treatment. CsA in association with TNF-alpha inhibitors (i.e., etanercept or adalimumab) has been safely and successfully used in a few series of PsA patients [79–81]. In these experiences, the addition of CsA was capable of inducing a notable benefit on cutaneous lesions as compared to biological therapy alone or associated with methotrexate. 

A retrospective analysis reviewed the results obtained with etanercept 50 mg once weekly and CsA, given at doses in the range of 3–5 mg/kg for two days per week, within an interval of 3-4 days (i.e., Monday and Thursday or Monday and Friday) [82]. This retrospective study involved 17 patients who required the combination therapy for different reasons: primary or secondary inefficacy of etanercept monotherapy, persistence of disabling cutaneous lesions at critical sites, or flare of psoriasis during etanercept treatment after interruption of efalizumab therapy. The addition of CsA was capable of inducing a relevant clinical benefit on skin lesions in a total of 12 patients. The combination treatment was well tolerated. Only a patient experienced one relevant side effect (repeated hypertensive crises) which caused CsA discontinuation after 2 months.

## 6. Conclusion

The available data consistently confirm that CsA is a very effective drug for the treatment of psoriasis, being capable of inducing a marked and prompt clinical response in the majority of treated patients. Dose and duration of CsA treatment are generally tailored to the patient's general characteristics and specific needs and should be adjusted throughout the treatment course in accordance with individual efficacy and tolerability. For treatment of plaque psoriasis and several other immunomediated skin disorders, CsA is generally used at a daily dosage of 2.5 up to 5 mg/kg. Intermittent regimens of 3–6 months are usually sufficient to achieve significant results in dermatological practice, while longer treatment is necessary in a minority of cases. It is however recommended to avoid continuous therapy with CsA for more than 2 years for safety reasons. Treatment regimens with pulse administration of CsA for a few days per week can be taken into consideration for the induction of psoriasis remission and as a maintenance therapy. Several studies have examined the role of CsA as a part of combination and rotational treatment strategies in psoriasis patients, and preliminary evidence supports the feasibility of short-term CsA addition as a rescue intervention to patients with psoriatic disease treated with biologicals.

Thanks to the huge clinical experience gathered after more than two decades of use in dermatological practice, CsA can be considered a manageable and flexible therapeutic tool for the treatment of psoriasis.

## Figures and Tables

**Table 1:** Main randomized studies with CsA for induction of remission in plaque-type psoriasis.

Reference	Pts	Baseline PASI	Treatment groups with CsA	Mean PASI improvement from baseline*	Other efficacy results
[9]	133	8–25	(1) 1.25 mg/kg/d; (2) 2.5 mg/kg/d	After 10 wks: (1) 27.2%; (2) 51%	PASI 75 in 63% of pts after other 12 wks with dosage adapted up to 5 mg/kg/d (mean dose: 2.99 mg/kg/d)

[10]	127	≥12	Starting dose: (1) 200 mg (BWI); (2) 2.5 mg/kg/d (BWD). Stepwise dose increase in case of nonresponse up to 300 mg (BWI) or 5 mg/kg/d (BWD)	After 12 wks: (1) 86%; (2) 87%	PASI 75 in 89.4% of total pts (BWI and BWD) after 12 wks

[11]	217	≥15	(1) 1.25 mg/kg/d; (2) 2.5 mg/kg/d. Dose doubling in case of nonresponse up to 5 mg/kg/d until achievement of response within 12–36 wks	—	Need of dose escalation in 27% of pts in group 2 and 68% in group 1. PASI 75 response rates within 12–36 wks according to CsA dose: (1) 1.25: 18%; 1.25–2.5: 43%; 1.25–5: 64%; (2) 2.5: 56%; 2.5–5: 72%

[12]	251	≥18	(1) 2.5 mg/kg/d; (2) 5 mg/kg/d	After 12 wks: (1) 69%; (2) 89%	Response (= PASI 75 or PASI < 8) after 12 wks: (1) 52%; (2) 92%

[13]	210	≥18	2.5 mg/kg/d (adjusted up to 5)	After 10 wks: 71.4%	At wk 10, mean dose: 3 mg/kg/d, without a need of dose change in 64% of pts. At 10 wks, PASI 60: 78.8%

[14]	309	≥15	(1) Sandimmun; (2) Neoral. In both groups, starting dose of 2.5 mg/kg/d with stepwise adjustments (up to 5 mg) according to efficacy or safety	After 16 wks: (1) 76.6%; (2) 79.6%	PASI 75 response rates: at 8 wks: (1) 38.2%; (2) 51.1%; at 16 wks: (1) 80.7%; (2) 87.3%

[7]	85	≥18	(1) 7.5 mg/kg/d; (2) 5 mg/kg/d; (3) 3 mg/kg/d	After 8 wks: (1) 71%; (2) 58%; (3) 39%	Pts clear or almost clear of lesions at 8 wks: (1) 80%; (2) 65%; (3) 36%

[15]	457	≥18	(1) 1.5 mg/kg/d; (2) 2.5–3 mg/kg/d; (3) 5 mg/kg/d	After 12 wks: (1) 35%; (2) 57%; (3) 86%	Response (= PASI 75 or PASI < 8) after 12 wks: (1) 24%; (2) 52%; (3) 88%

[16]	88	≥8	3 mg/kg/d (up to 5 in case of nonresponse)	After 16 wks: 72%	PASI 75 after 16 wks: 71%

[17]	84	No limit	3 mg/kg/d (up to 5 in case of nonresponse)	After 12 wks: 72%;	PASI 75 after 12 wks: 58%

Many of these trials were controlled versus placebo or other active systemic drugs (i.e., etretinate or methotrexate), but the results related to the other treatment groups are omitted.

*Results are distinguished according to the treatment group (see the corresponding number in the related column).

BWD: body weight dependent; BWI: body weight independent; CsA: cyclosporine; d: day; PASI: Psoriasis Area and Severity Index; PASI 60: PASI improvement of at least 60% from baseline; PASI 75: PASI improvement of at least 75% from baseline; pts: patients.

**Table 2:** Main studies with CsA after induction of remission in moderate-to-severe plaque-type psoriasis.

Reference	Pts	Study design and CsA treatment details in responders after induction therapy with CsA	Synopsis of efficacy results
[44]	51	Continuous regimen (at the lowest effective dose) or intermittent treatment (discontinuation with 12-week courses in the event of relapse) for 9 months	PASI 75 response rates significantly higher with continuous treatment (92% versus 62%). Median effective maintenance daily doses: 3 (2.5–3.8) for intermittent schedule and 1.8 (0.7–3) mg/kg for continuous regimen.

[45]	31	Continuous regimen with 0.5–3 mg/kg/d or intermittent regimen, with dose tapering off and use of topical steroids, when necessary, until relapse (mean follow-up: 55.9 months)	Decrease of PASI by more than 70% from baseline with both regimens. Better overall control of psoriasis with continuous therapy.

[36]	217	1.25, 2.5, or 5 mg/kg/d for 6 to 30 months	Maintenance of clinical improvement during maintenance therapy in pts achieving the PASI 75 with their individual dose of CsA. 12.5% of the pts maintained on 1.25 mg/kg/d without loss of efficacy.

[12]	251	2.5 mg/kg/d (escalated to 5 mg in case of relapse) for 12 wks	Maintenance of response until month 10 in 68–77% of pts on 2.5 mg/kg/d

[46]	181	1.5 or 3 mg/kg/d (or placebo) for 6 months	Psoriasis relapse in 42% of pts on 3 mg/kg/d versus 84% of pts on placebo. Median time to relapse: 6 wks with both placebo and CsA 1.5 mg/kg/d

[47]	61	1.5 or 3 mg/kg/d (or placebo) for maximum 16 wks	Mean time to relapse: 12 wks in the 3 mg/kg, 9 wks in the 1.5 mg/kg, and 7 wks in the placebo groups, without differences between the latter two groups. Absence of relapse in 57% of pts on 3 mg/kg versus 21% and 5% of the 1.5 mg/kg and placebo groups, respectively.

[6]	400	Intermittent treatment up to four courses (2.5–5 mg/kg/d for a maximum of 12 wks) within 1 year	Absence of relapse in 45% of pts 16 wks after stopping treatment and in 31% after 24 wks.

CsA: cyclosporine; d: day; PASI: psoriasis area and severity index; PASI 75: PASI improvement of at least 75% from baseline; pts: patients.
